# Induced pluripotent stem cell models of lysosomal storage disorders

**DOI:** 10.1242/dmm.029009

**Published:** 2017-06-01

**Authors:** Daniel K. Borger, Benjamin McMahon, Tamanna Roshan Lal, Jenny Serra-Vinardell, Elma Aflaki, Ellen Sidransky

**Affiliations:** Medical Genetics Branch, National Human Genome Research Institute, National Institutes of Health, Bethesda, MD 20892, USA

**Keywords:** Gaucher disease, IPSC models, Lysosomal enzymes, Lysosomal storage disorders, Neurodegeneration

## Abstract

Induced pluripotent stem cells (iPSCs) have provided new opportunities to explore the cell biology and pathophysiology of human diseases, and the lysosomal storage disorder research community has been quick to adopt this technology. Patient-derived iPSC models have been generated for a number of lysosomal storage disorders, including Gaucher disease, Pompe disease, Fabry disease, metachromatic leukodystrophy, the neuronal ceroid lipofuscinoses, Niemann-Pick types A and C1, and several of the mucopolysaccharidoses. Here, we review the strategies employed for reprogramming and differentiation, as well as insights into disease etiology gleaned from the currently available models. Examples are provided to illustrate how iPSC-derived models can be employed to develop new therapeutic strategies for these disorders. We also discuss how models of these rare diseases could contribute to an enhanced understanding of more common neurodegenerative disorders such as Parkinson’s disease, and discuss key challenges and opportunities in this area of research.

## Abstract (precis)

**Summary:** This Review discusses how induced pluripotent stem cells (iPSCs) provide new opportunities to explore the biology and pathophysiology of lysosomal storage diseases, and how iPSCs have illuminated the role of lysosomes in more common disorders.

## Introduction

There are over 50 types of lysosomal storage disease, a class of inherited metabolic disease caused by the absence or deficiency of a lysosomal protein. Most lysosomal storage diseases (LSDs) result from mutations in metabolic enzymes that are active in the lysosome, although a handful are caused by defects in lysosomal transport or vesicular trafficking (Boustany, 2013; Feng et al., 2002; Ward et al., 2000). Regardless of the function of the mutated gene, LSDs are universally characterized by the intracellular accumulation of undigested storage material, although the composition of this material varies between the LSDs. The deficiency of functional protein and the subsequent accumulation of storage material can impair normal lysosomal function in affected cells and interrupt a diverse array of cellular activities (Ballabio and Gieselmann, 2009). The phenotypic consequences in patients are extremely varied, ranging from asymptomatic or sub-clinical manifestations; to chronic visceral, musculoskeletal or immunological disease, to lethal acute neuronopathic disease. Even within a given LSD, there can be vast phenotypic heterogeneity, with many LSDs exhibiting both early- and late-onset forms (Boustany, 2013).

Individual LSDs are extremely rare disorders, but taken together, they are thought to affect up to 1 in 4000 live births (Al-Jasmi et al., 2013; Applegarth et al., 2000; Meikle et al., 1999; Pinto et al., 2004; Poorthuis et al., 1999; Poupětová et al., 2010). Furthermore, due in part to the multi-organ nature of many of these diseases, and because of a continuing lack of truly effective treatments for many LSDs, these diseases are often characterized by high mortality and morbidity (Stone and Sidransky, 1999). Current therapies are extremely costly and are often lifelong treatments. LSDs therefore constitute a significant burden on affected individuals and their families and on healthcare systems as a whole, and LSDs have long been a major focus of rare disease research. Moreover, a growing appreciation of the role of lysosomal dysfunction in aging and age-related neurodegenerative disorders has led to a recent surge of interest in LSDs. These factors have combined to help fuel the application of a number of emerging biotechnologies towards LSD research. However, there has been mixed success in generating suitable animal models of LSDs for research in this field (Farfel-Becker et al., 2011; Lawson and Martin, 2016; Pastores et al., 2013), and hence investigators have directed efforts toward developing alternative disease models.

One technology in particular – the development of induced pluripotent stem cells (iPSCs) – has been broadly adapted by investigators researching the LSDs, and human iPSC lines are already contributing significantly to our understanding and treatment of these rare diseases. Here, we review the use of human iPSCs in LSD research, highlighting the strategies that have been used to generate iPSCs and iPSC-derived cell models and to evaluate their relative success in accurately phenocopying the human disease. The benefits of these cells in untangling disease etiology and developing novel therapeutics are discussed, as well as the limitations. We also briefly highlight how the insights gleaned from studying LSDs using these cellular models could contribute to a better understanding of more common neurodegenerative diseases.

## Induced pluripotent stem cells: an overview

In 2006, Shinya Yamanaka and his colleagues at Kyoto University reported that by forcing expression of four genes – *OCT3/4*, *SOX2*, *KLF4* and *MYC* (collectively known as OSKM) – via retroviral transduction, they were able to convert murine fibroblasts into fully pluripotent stem cells. The profile and potency of these murine iPSCs were similar to those in embryonic stem cells (Takahashi and Yamanaka, 2006). The following year, three papers – one by Yamanaka's group (Takahashi et al., 2007) and one by George Daley's group (Park et al., 2008a), both using the OSKM cocktail, and a third by James Thomson's group (Yu et al., 2007) using *OCT3/4*, *SOX2*, *NANOG* and *LIN28* (OSNL) – showed that the same basic technique used in mice could also be employed to generate iPSCs from human somatic cells. Since then, numerous advances have been made in identifying new factors that induce reprogramming, which now include RNAs and small molecules, new modes of introducing the necessary factors to cells, and new cell types that can be reprogrammed (Table 1). These discoveries have done much to inform our understanding of how stem cells achieve and maintain pluripotency. Recent work clearly demonstrates how iPSC-derived cells are a remarkable tool for research of human diseases (see Box 1). These advantages have made iPSC-derived cell models a natural choice for studies of the LSDs, as discussed below.

**Table 1.**
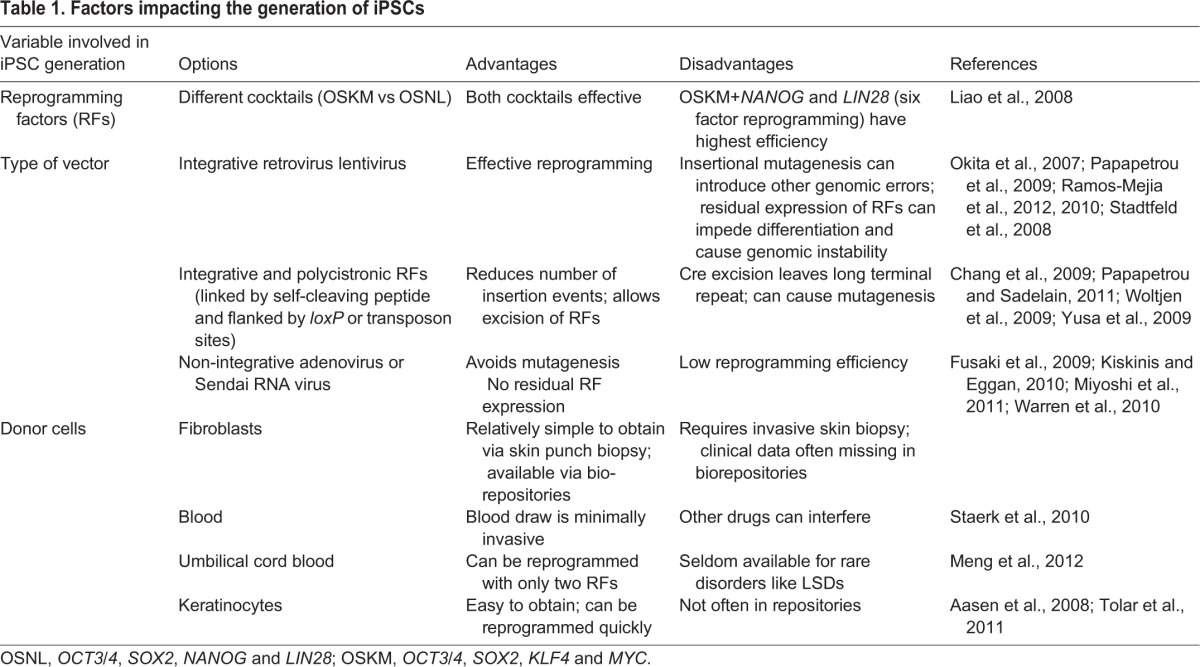
**Factors impacting the generation of iPSCs**

**Box 1: Advantages of iPSC technology**

iPSCs offer an effective means of developing *in vitro* human cellular models for diseases which previously lacked such models

They enable the generation of certain cell types that are difficult or impossible to obtain directly from humans

Sufficient numbers can be generated to perform cell-based experiments and drug screens

**Fig. 1.**
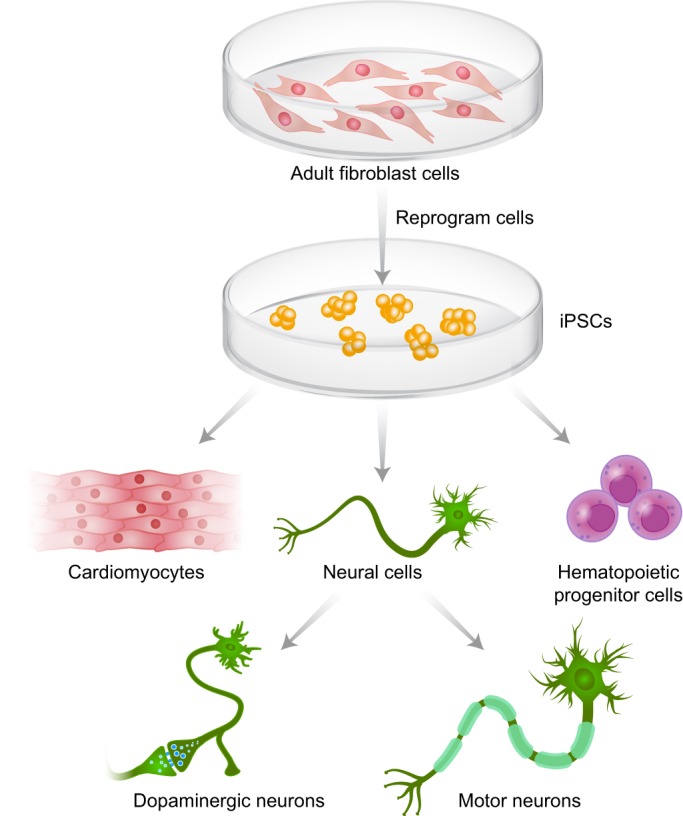
**Patient-derived fibroblasts can be reprogrammed into iPSCs and then differentiated into different cellular lineages.** Adult fibroblasts are reprogrammed into iPSCs, which can be differentiated into different lineages. Neural precursor cells can be further differentiated into specific types of neurons such as dopaminerigic neurons or motor neurons.

iPSCs can be derived from a plethora of cell types and then differentiated into different cellular types (Fig. 1)

Once generated, they can be frozen, thawed and expanded, thereby providing an unlimited supply of cells for research

iPSCs are free of the controversy and legal limitations facing embryonic stem cell use

## iPSC models of LSDs

**Fig. 2.**
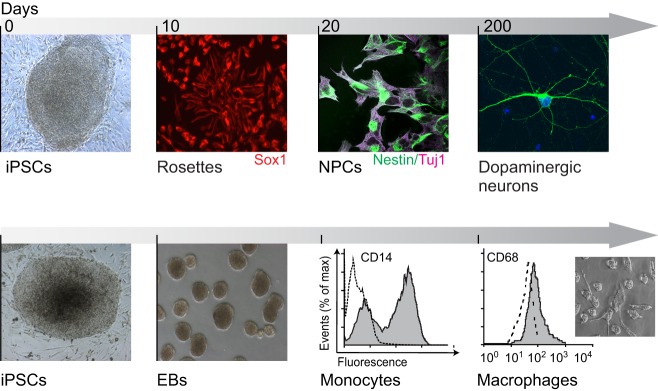
**Differentiation of iPSCs to neurons and macrophages.** Representative fluorescent microscopy images illustrating how rosettes, a distinct form of neuronal stems cells that stain positive for Sox1, are generated from the iPSCs (top row). Rosettes are then differentiated into neuronal progenitor cells (NPCs), which stain positive for the neuronal markers Nestin and Tuj1. Further differentiation into mature dopaminergic neurons, which can be visualized by staining with tyrosine hydroxylase, can take as long as 200 days. To make monocytes and macrophages (bottom row), the first stage is generation of embryoid bodies (EBs, visualised here by phased light microscopy), which are spherical aggregates that recapitulate many features of early embryogenesis. Monocytes, which can be identified by the immunological marker CD14, can then be separated by a fluorescence-activated cell sorter and harvested. Finally, CD14-positive monocytes can be differentiated into CD68-postive macrophages. The plots show the separation of CD14- and CD68-positive cells based on fluorescence intensity, and the smaller panel provides a representative light microscopy image of macrophages.

Cells of the neuronal and hematopoietic lineages are the usual differentiation targets for iPSC-derived models of LSDs (28 of 39 studies pursuing differentiation; see Table 2) because these are the cells most often affected by these diseases (Figs 1 and 2). Although murine iPSC lines have been derived from five mouse models of LSDs (Kawagoe et al., 2011; Meng et al., 2010; Ogawa et al., 2013), human iPSCs and iPSC-derived cell models, which have been generated for at least 11 LSDs (Table 2), have become the focus of the field, as they more closely mimic the human disease. As discussed below, human iPSC models of LSDs are already contributing to our understanding and treatment of these rare diseases.

**Table 2.**
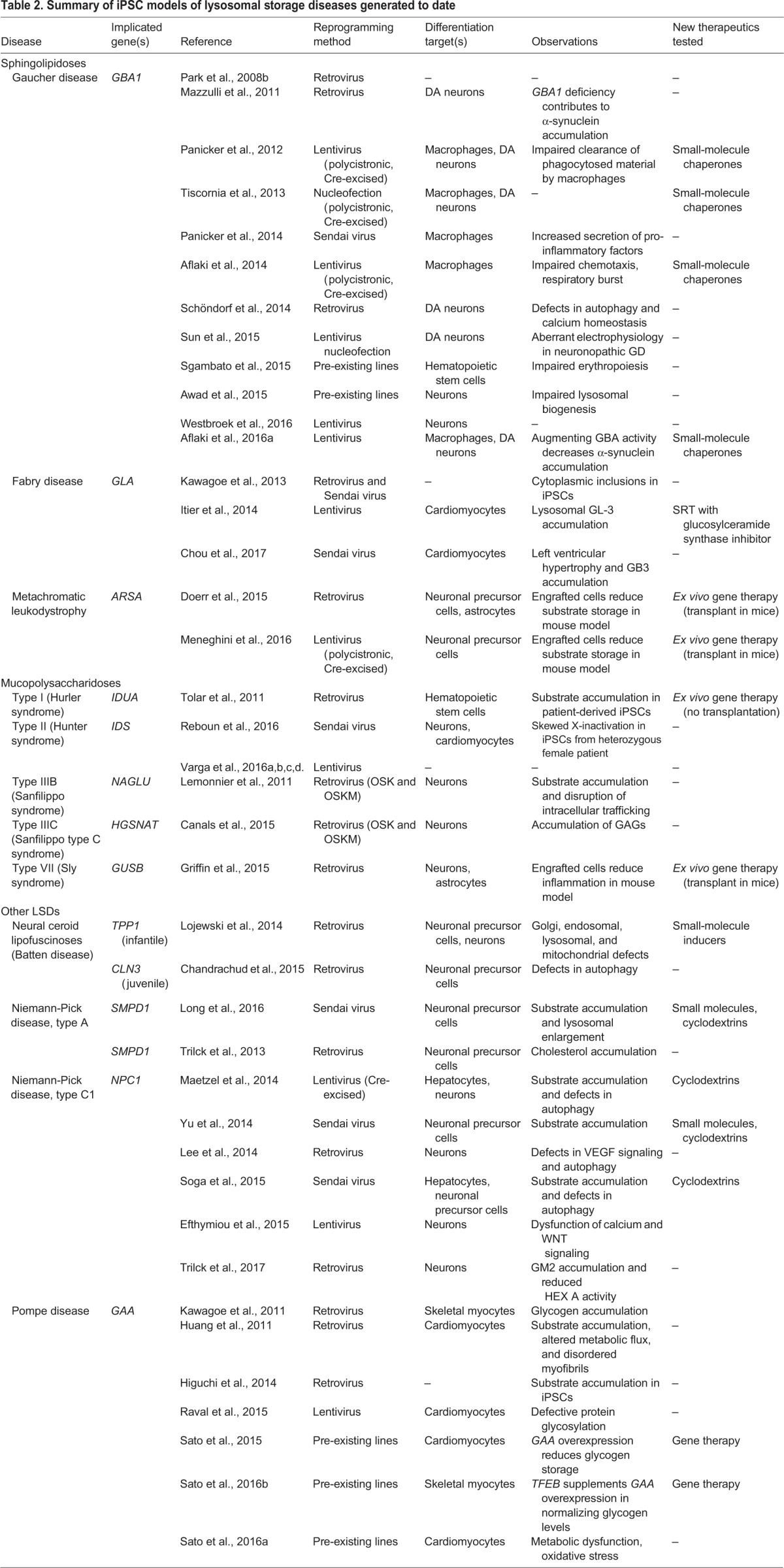
**Summary of iPSC models of lysosomal storage diseases generated to date**

### Gaucher disease

Gaucher disease (GD) is a recessive disorder caused by mutations in *GBA1*, resulting in a deficiency in a lysosomal hydrolase named glucocerebrosidase (Mistry et al., 2017). Usually, this deficiency leads to glycolipid accumulation in macrophages, and manifests in hematological, visceral and skeletal symptoms, with a variable degree of severity. GD is classified into three types: non-neuronopathic (type 1), acute lethal neuronopathic (type 2), and chronic neuronopathic (type 3), and more severe mutations are associated with neuronopathic manifestations. Furthermore, population studies have established that individuals with *GBA1* mutations, both carriers and affected individuals, are at an increased risk of developing Parkinson’s disease, an age-related neurodegenerative disorder, as well as other Lewy body disorders (Nalls et al., 2013; Sidransky et al., 2009). The discovery of this link between *GBA1* and Parkinson's disease has played a large part in a recent explosion of GD research.

However, both seasoned GD researchers and those new to the field have been limited by a dearth of effective models for studying both GD and *GBA1*-associated Parkinson's disease. Mouse models for GD have been developed, but none faithfully recapitulate the features of the disease seen in humans (Farfel-Becker et al., 2011) Human cell lines have also been of limited use. Fibroblasts from patients with GD have long been used to study the disease, but these cells do not store the implicated glycolipids and do not show obvious signs of pathology (Saito and Rosenberg, 1985). Applying conduritol β-epoxide (CBE), an irreversible inhibitor of glucocerebrosidase, to a common human monocytic cell line such as THP-1 or to SH-SY5Y cells (a human neuroblastoma cell line used to model neurons) leads to glycolipid accumulation in these cell lines, and this approach has been used to provide *in vitro* models for GD (Hein et al., 2007; Prence et al., 1996). However, it is difficult to generalize findings in immortalized cell lines to cells *in vivo*. The limited options for studying GD in the laboratory has directed attention toward iPSCs.

As macrophages are the primary cell type affected in all GD types, they are a natural choice for studies of this disease (Fig. 2). It has generally been accepted that glycolipid-engorged macrophages drive the systemic inflammation commonly seen in GD, indicated by elevated inflammatory factors in the blood of affected individuals (Pandey and Grabowski, 2013). Differentiating iPSCs derived from patients with type 1 GD into macrophages, Panicker et al. (2014) found that these GD iPSC-derived macrophages secrete greatly elevated levels of pro-inflammatory factors, including interleukin (IL)-10, IL-6, IL-1β, and tumor necrosis factor (TNF)-α, when challenged with lipopolysaccharide, a common pro-inflammatory stimulus. GD iPSC-derived macrophages also exhibit elevated secretion of chitotriosidase, an antifungal factor that is often used as a biomarker for the severity of visceral GD symptoms (Panicker et al., 2014). This pro-inflammatory profile of GD macrophages has been further explored using primary human macrophages (Aflaki et al., 2016b). In addition, GD iPSCs were reprogrammed to macrophages, which exhibited deficient glucocerebrosidase activity, increased glycolipid storage and impaired translocation of glucocerebrosidase to the lysosome. Comparing these cells with primary macrophages made from monocytes isolated from the same patients, a similar phenotype, including impaired chemotaxis and reactive oxygen species production (ROS) was observed, demonstrating the ability of the iPSC-derived cells to phenocopy the primary cells. (Aflaki et al., 2014). GD iPSCs have also been used to explore hematopoiesis in GD, leading to the conclusion that glucocerebrosidase deficiency directly impairs hematopoietic development (Sgambato et al., 2015).

In order to study the neuropathology and the role of *GBA1* in parkinsonism, GD iPSCs have also been differentiated into neurons. The first such study by Mazzulli et al. (2011) utilized a fibroblast cell line, derived from the cells of a 20-year-old male with GD, to make dopaminergic (DA) neurons. Subsequently, two other groups also differentiated GD iPSCs to neurons that stain positive for tyrosine hydroxylase (TH), a marker for DA neurons (Panicker et al., 2012; Tiscornia et al., 2013). In addition, these studies demonstrated that cells from infants with type 2 GD could be successfully differentiated despite their profound deficiency of glucocerebrosidase. A more recent study showed that iPSC-derived neurons from patients with GD exhibit abnormal lysosomal function and altered lysosomal biogenesis (Awad et al., 2015).

iPSCs, neuronal precursor cells (NPCs) and neurons generated from an infant with type 2 GD all have similar degrees of glucocerebrosidase deficiency compared with the original patient fibroblasts (Sun et al., 2015). These cells had increased levels of both glucosylsphingosine and glucosylceramide – the enzyme substrates. Functional studies utilizing whole-cell patch-clamping of the type 2 GD iPSC-derived neurons demonstrated excitation characteristics of neurons, and, interestingly, these cells showed reductions in action potential amplitudes and sodium and potassium currents. The authors suggest that the abnormal electrophysiological properties observed in these neurons provide new clues into the pathogenesis of the neuronopathic phenotype in Gaucher disease (Sun et al., 2015).

In another study, Aflaki et al. (2016a) examined differentiated neurons from iPSCs from patients with type 1 GD with and without Parkinson's disease, and from a patient with GD2. The neurons had deficient glucocerebrosidase, stored glucosylceramide and glucosylsphingosine and co-localization studies revealed greatly reduced levels of lysosomal glucocerebrosidase in the DA neurons indicating an appropriate Gaucher phenotype (Aflaki et al., 2016a). Overall, these studies indicate that iPSC-based models of Gaucher disease successfully recapitulate hallmarks of this LSD.

### Pompe disease

Pompe disease is an autosomal recessive LSD caused by mutations in *GAA*, the gene coding for the glycolytic enzyme α-glucosidase, which lead to glycogen accumulation in myocytes (Dasouki et al., 2014). Pompe disease is divided into two major types based on age of onset: an infantile-onset form where glycogen accumulation occurs primarily in cardiomyocytes and a later-onset form where glycogen accumulation is primarily restricted to skeletal muscle (Chan et al., 2017). Like many other LSDs, Pompe disease research has been constrained by a lack of physiologically relevant models. Although Pompe disease mouse models exhibit cellular manifestations similar to those in humans, their overall clinical phenotype differs greatly from that encountered in patients (Lim et al., 2014).

The development of iPSCs from patients with Pompe disease has been met with variable results. Huang et al. (2011) were the first to attempt reprogramming fibroblasts from patients with Pompe disease. Initially, there were problems working with the enzyme-deficient cells, which may have resulted from metabolic impediments to reprogramming and differentiation in the background of GAA deficiency. The group were eventually able to successfully recover reprogrammed control iPSCs after restoring α-glucosidase activity via lentiviral delivery of inducible wild-type *GAA* prior to OSKM transduction. Notably, several of the patient-derived iPSC clones subsequently recovered were not transfected with *GAA­­*-containing vectors, with the authors hypothesizing that exogenous enzyme from nearby α-glucosidase-expressing cells was sufficient to overcome any metabolic barrier to reprogramming (Huang et al., 2011). However, two subsequent studies on Pompe disease described the successful generation of patient-derived iPSC clones made in the absence of exogenous enzyme (Higuchi et al., 2014; Raval et al., 2015).

Raval et al. (2015) reprogrammed fibroblasts from patients with infantile-onset Pompe disease and differentiated them into cardiomyocytes. Although these cells exhibited no α-glucosidase activity and the lysosomes were engorged with glycogen, contractility and autophagy in these cells were not impaired and functionally, Pompe disease cells were indistinguishable from controls. Nonetheless, the cardiomyocytes did have aberrant glycan processing in some proteins, suggesting that this may play a role in the development of the cardiomyopathy characteristic of this disorder.

Another study focused on iPSC-derived cardiomyocytes from patients with late-onset Pompe disease, and also confirmed the accumulation and storage of glycogen in lysosomes (Sato et al., 2015). The authors then partially corrected the defect using lentiviral *GAA*, resulting in enhanced α-glucosidase activity and decreased glycogen accumulation (Sato et al., 2015). In an attempt to further enhance α-glucosidase activity in skeletal muscle derived from Pompe iPSCs, they introduced the gene encoding transcription factor EB (TFEB), a master regulater coordinating the expression of lysosomal hydrolases, membrane proteins and genes involved in autophagy. *GAA* and *TFEB* together yielded further biochemical improvement in the form of a reduction of the glycogen stores in muscle cells and improved enzymatic activity in cells (Sato et al., 2016b). This finding implicated abnormal lysosomal biogenesis in the muscular pathology of Pompe disease. The same group also performed metabolomic profiling of the cells, which demonstrated that oxidative stress and mitochondrial dysfunction are associated with the disorder. The work was then replicated in iPSCs derived from a genetically engineered murine Pompe model, and this confirmed the role of oxidative stress in skeletal and cardiac dysfunction in this disorder (Sato et al., 2016a). Furthermore, the authors found that the nuclear factor, erythroid 2 (NF-E2), which plays a key role in combating oxidative stress, is downregulated in Pompe cardiomyocytes and skeletal muscle, implicating an impaired anti-oxidative stress response mechanism in the pathophysiology of disease.

### Fabry disease

Fabry disease is an X-linked recessive LSD that results from deficient or absent activity of the enzyme α-galactosidase A, which leads to the progressive lysosomal accumulation of globotriaosylceramide (Gb3) in a variety of cell types, such as cardiomyoctes (Schiffmann and Ries, 2016; Ranieri et al., 2016). This systemic Gb3 accumulation eventually leads to devastating renal, cardiac and cerebrovascular dysfunction.

The first Fabry disease iPSCs that were generated exhibited ultrastructural features typically seen in Fabry disease, including membranous cytoplasmic bodies (Kawagoe et al., 2013). This led the authors to speculate that differentiating Fabry iPSCs into other lineages could be challenging. However, iPSCs were later generated from fibroblasts isolated directly from patients with Fabry disease (Itier et al., 2014). These iPSCs exhibited no detectable Gb3 and could differentiate into cardiomyocytes. Over time, Gb3 accumulated in the lysosomes of these cardiomyocytes, mimicking the phenotypic changes found in cardiac tissue from patients with Fabry disease. Using the cardiomyocytes as a model, it was demonstrated that ibiglustat, a glucosylceramide synthase inhibitor being developed as a substrate reduction therapy for Fabry disease, prevented Gb3 accumulation and eventually cleared lysosomal Gb3 (Itier et al., 2014). Thus, ibiglustat could be a promising therapeutic strategy for this lysosomal storage disease.

### Metachromatic leukodystrophy

Metachromatic leukodystrophy (MLD), is an autosomal recessive disorder of lipid metabolism characterized by the deficient activity of the lysosomal enzyme arylsulfatase A (ASA), resulting in deficient degradation of galactosylceramide-3-*O*-sulfate (sulfatide) and galactosylsphingosine-3-*O*-sulfate (lysosulfatide) (Gieselmann, 2008). At the cellular level, the disease is characterized by impaired sphingolipid metabolism and the resulting accumulation of sulfatide. Progressive accumulation of sulfatide in the myelin-producing cells causes destruction of white matter in both the central and peripheral nervous systems, driving progressive deterioration of intellectual functions and motor skills, including the ability to walk. There are three clinical subtypes of this disorder: late-infantile, juvenile and adult forms. Symptoms seen in individuals who are affected include peripheral neuropathy, incontinence, seizures, paralysis, a loss of the ability to speak, and visual and hearing loss. Patients can eventually become unresponsive to their surroundings (Gieselmann, 2008).

Two studies have demonstrated successful generation and differentiation of iPSCs for MLD. In a study by Doerr et al. (2015), MLD patient-derived iPSCs were differentiated into self-renewing neuroepithelial stem cells and astroglial progenitors, which were then used to evaluate cell-based ARSA replacement. Transplantation of ARSA-overexpressing precursors into ARSA-deficient mice resulted in significantly reduced sulfatide levels (Doerr et al., 2015). Recently, the differentiation of MLD patient fibroblasts into iPSC models was performed by Meneghini et al. (2017). The patient-derived iPSCs were differentiated into neural stem cells, which shared molecular, phenotypic and functional features with fetal-derived MLD neural stem cells. Using lentiviral vectors, MLD iPSCs were efficiently transduced, achieving supraphysiological ARSA activity, which increased further after neural differentiation. A significant decrease in sulfatide storage was also observed when ARSA-overexpressing cells were used (Meneghini et al., 2017). This study enhances our understanding of the CNS pathology in MLD, and suggests that ultimately, cell transplantation might provide both enzymatic reconstitution and replacement of damaged or lost cells.

### Neuronal ceroid lipofuscinoses

Neuronal ceroid lipofuscinoses (NCLs), also referred to collectively as Batten disease, are a group of extremely rare and fatal neurodegenerative LSDs. These diseases are characterized by intracellular accumulation of autofluorescent lipofuscin, a fatty lipopigment, in both neurons and peripheral tissues (Mole and Cotman, 2015). To date, mutations in 14 genes have been identified as being potentially causative for NCLs, and there are several NCL subtypes based on the mutated gene, age of onset, and the severity of neurological defects such as progressive dementia, seizures and visual failure (Mole and Cotman, 2015).

A study by Lojewski and co-workers in 2014 generated the first NCL iPSCs, using fibroblasts derived from two patients with late-infantile NCL linked to mutations in *TPP1* (tripeptidyl peptidase 1), and four patients with juvenile NCL and mutations in *CLN3*. *TPP1* encodes a member of the sedolisin family of serine proteases and *CLN3* encodes a protein involved in lysosomal function. These patient-derived iPSCs were differentiated into neuronal tissue. As expected, abnormalities in the endosomal-lysosomal system were detected in the patient iPSCs, but the authors noted that disease-subtype-specific lysosomal storage was only evident in their differentiated neuronal derivatives. They were able to correct the abnormalities in these cells by overexpressing adenovirus vector-delivered wild-type *TPP1* or *CLN3.* These iPSC-derived neural progenitor cells were also used to screen potential pharmacological modulators of the *CLN2* encoded protein. The screen demonstrated the utility of patient-derived iPSCs as a platform for testing new therapeutic candidates. Two lipid-lowering drugs were identified – fenofibrate and gemfibrozil. The patient with the NCL-linked *TPP1* mutation was treated with these compounds, resulting in a small increase in both *TPP1* levels and enzymatic activity. This work further illustrates the value of iPSC-derived human neuronal models for NCL drug discovery and evaluation.

### Niemann-Pick type C disease

Niemann-Pick type C disease (NP-C) is an autosomal recessive neurovisceral atypical LSD. Mutations in *NPC1* and *NPC2* lead to impaired intracellular transport of cholesterol and glycolipids, which ultimately causes accumulation of these lipids in cells (Vance, 2006). Both NPC1 and NPC2 proteins are catalysts that mobilize the cholesterol within the multivesicular environment of the late endosome. Children affected by NP-C present primarily with visceral symptoms such as hepatosplenomegaly (enlargement of the liver and spleen) followed by progressive intellectual and neurological deterioration. Those who present in adulthood often develop psychiatric problems, including depression and psychosis (Evans and Hendriksz, 2017).

Hepatocyte-like cells and neural progenitors derived from the iPSC lines generated from patient-derived fibroblasts displayed cholesterol accumulation and impairment of autophagy and ATP production (Soga et al., 2015), indicating that these cells do phenocopy the human disease. Soga et al. (2015) also showed that a new compound, 2-hydroxypropyl-γ-cyclodextrin, reduced cholesterol accumulation and restored the observed abnormalities in the patient-derived NPC iPSCs, demonstrating the utility of this model for evaluating new candidate drugs.

In another study, patient-derived NP-C iPSC neurons were found to have abnormal vascular endothelial growth factor (VEGF) levels and altered sphingolipid metabolism, thus recapitulating features of the disease *in vivo* (Lee et al., 2014). The neurons also demonstrated inhibition of autophagosome-lysosome fusion when compared with wild-type neurons. Treatment with VEGF appeared to ameliorate this defect in autophagy by correcting the sphingolipid abnormalities, indicating that VEGF could be a therapeutic candidate for Niemann-Pick type C disease.

Bergamin et al. (2013) successfully generated a human neuronal model of NP-C by inducing neuronal differentiation of multipotent adult stem cells (MASCs) isolated from patients with NP-C and controls. In the MASCs, massive lysosomal accumulation of cholesterol was observed only in those isolated from patients with NP-C. Upon neural differentiation, intracellular accumulation of unesterified cholesterol and GM2 ganglioside were observed in the NP-C neurons, resulting in morphological differences that distinguished the diseased cells from those derived from healthy donors. It is likely that these promising iPSC models will soon be used to explore the pathophysiology of NP-C.

### The mucopolysaccharidoses

The mucopolysaccharidoses (MPSs) are a heterogeneous group of LSDs that are clinically characterized by progressive dysfunction in multiple organ systems and reduced life expectancy (Coutinho et al., 2012). Apart from MPS II (also known as Hunter Syndrome), which is inherited in an X-linked manner, the MPSs are autosomal recessive diseases. Individuals with MPSs are typically healthy at birth, but during early childhood they experience onset of symptoms that include deterioration of skeletal, joint, airway and cardiac tissue; impaired hearing and vision; and, in some MPSs, cognitive impairment. There are nine subtypes of MPS described to date, each caused by a deficiency in a lysosomal enzyme required for glycosaminoglycan (GAG) degradation. The result of this deficiency is accumulation of partially degraded GAG within lysosomes and elevated levels of GAG fragments in the urine, blood and cerebral spinal fluid (Coutinho et al., 2012).

IPSCs have been generated from patients with MPS IH (Hurler syndrome), which is caused by the deficiency of α-L-iduronidase. The study indicated that the deficient enzyme is not required for stem cell renewal (Tolar et al., 2011). The iPSCs showed lysosomal storage defects characteristic of MPS IH and could be differentiated to both hematopoietic and non-hematopoietic cells. The authors demonstrated that when the differentiated cells were gene-corrected with virally delivered α-L-iduronidase, the specific epigenetic profile associated with de-differentiation of MPS IH fibroblasts into MPS-iPSCs was maintained, highlighting the potential of these cells to generate autologous hematopoietic grafts devoid of immunologic complications (Tolar et al., 2011). Hematopoietic cell transplantation is currently being performed as a life-saving treatment for MPS IH. However a suitable hematopoietic donor is not found for all affected individuals, and the therapy is associated with significant morbidity as well as mortality (Aldenhoven et al., 2008). The potential to generate gene-corrected autologous stem cells could potentially provide a more optimal graft for transplantation, avoiding current complications.

As it is an X-linked disorder, MPS II manifests almost exclusively in males; however, an iPSC model has been generated from a symptomatic female with a heterozygous mutation in the *IDS* (iduronate 2-sulfatase) gene (Reboun et al., 2016). This gene encodes a member of the sulfatase family of proteins, which is involved in the lysosomal degradation of heparan sulfate and dermatan sulfate. iPSCs generated from the patient's peripheral blood demonstrated characteristic pluripotency markers and deficient iduronate 2-sulfatase activity. This study reported that X-inactivation, analyzed at three X-chromosome loci, showed extreme skewing in two of the patient's cell types, favoring exclusive expression of the mutated allele. iPSCs derived principally from males affected by MPSII have also been successfully generated by Varga et al., (2016a,b,c,d).

In their initial attempt at generating iPSCs for MPS IIIB (Sanfillipo syndrome type B), Lemonnier et al. (2011) were unsuccessful, and the authors speculated that accumulation of improperly metabolized GAG in patient-derived iPSCs interfered with growth factor signaling. Co-culture of the patient-derived iPSCs with feeder cells secreting α-N-acetylglucosaminidase, showed that the deficient enzyme in MPS IIIB, was necessary to expand the resulting iPSCs (Lemonnier et al., 2011).

iPSC lines have also generated from two patients with MPS IIIC (Sanfilippo syndrome type C) (Canals et al., 2015). Neurons derived from these lines recapitulated features of the disease, including low acetyl-CoA α-glucosaminide N-acetyltransferase activity, accumulation of GAG, and an increase in lysosome size and number, which was not seen in genetically corrected patient-specific iPSC-derived cultures. Furthermore, the authors observed early defects in neuronal activity, neuronal-wide degradation, and altered effective connectivity in the patient-derived cells. Since the mechanism underlying the brain dysfunction and behavioral phenotype in this disorder are poorly understood, the identification of these early functional phenotypes provide new insight into disease pathogenesis. Furthermore, the model has utility for drug development (Canals et al., 2015).

Another study of mucopolysaccharidoses used human iPSCs generated from patients with MPS VII (Sly syndrome). MPS VII iPSCs were differentiated into neuronal precursor cells and then transplanted into a well-characterized mouse model of the disease (Griffin et al., 2015). The patient-derived neural stem cells engrafted along the rostrocaudal axis of the CNS primarily within white matter tracts, surviving around four months. Genetically corrected iPSC-derived neural stem cells were transplanted into the striatum of adult post-symptomatic MPSVII mice, resulting in a reversal of neuropathology in a zone surrounding the grafts (Griffin et al., 2015). This study suggested the potential of *ex vivo* gene therapy in the brain for LSDs, discussed further below.

## A therapeutic revolution for the LSDs?

Until relatively recently, therapeutic options for LSDs have been largely limited to palliative care and physical therapy. Bone marrow transplant has been attempted as a means to treat a handful of the LSDs, but transplant-associated morbidity and mortality and the failure of this procedure to alleviate neurological manifestations in some LSDs have limited its wider application (Rovelli, 2008). This made the development of enzyme replacement therapy (ERT), which is currently available or in clinical trials for eight LSDs (Ries, 2017), a revolution in the field of LSDs. ERT involves intravenous infusion of the deficient enzyme, with the aim of clearing stored material and restoring normal lysosomal function in affected cells. ERT is effective in preventing or reversing visceral, cardiovascular, musculoskeletal, and even peripheral neurological manifestations of those diseases for which it is available (Barton et al., 1991; Schiffmann et al., 2003, 2001; Winkel et al., 2004). However, the infused enzymes are unable to cross the blood-brain barrier, and thus have little impact on brain phenotypes in neuronopathic LSDs. Furthermore, it is an inconvenient and extremely expensive treatment, requiring infusions at regular intervals for the remainder of the patient's life at a cost upwards of US$200,000 per year (Kanters et al., 2014; van Dussen et al., 2014).

Another therapeutic approach, substrate reduction therapy (SRT), involves the administration of small-molecule inhibitors aimed at reducing the synthesis of storage material. To date, SRT has demonstrated only mixed success in managing neurological symptoms of LSDs. One SRT drug, miglustat, has shown some promise in slowing neurological decline in Niemann-Pick type C disease, but the same drug (and a second SRT, elglucerase) showed no impact on the neurological symptoms in GD (Patterson et al., 2007; Schiffmann et al., 2008; Poole, 2014; Shayman, 2010). Other SRT drugs are currently in clinical trials for Pompe disease, Gaucher disease and Niemann-Pick C (Parenti et al., 2015).

These realities, paired with recent technological developments, have pushed the development of new and improved treatment modalities to the forefront of LSD research. Modifications are being developed to allow enzymes infused intravenously to cross the blood-brain barrier and enter neurons and glial cells (Grubb et al., 2008; Sorrentino et al., 2013). Gene therapy and corrective stem cell therapies are also being investigated in animal models as potential treatments for severe LSDs, with a particular focus on lethal neuropathic LSDs (Sands and Haskins, 2008). Alongside these developments, new approaches using small-molecule pharmacological chaperones have attracted much attention as a potential therapy (Parenti et al., 2015).

### iPSC-based therapies

One exciting development in iPSC research for LSDs is the possibility of *ex vivo* gene therapy, especially as a means of treating neuronal manifestations of these diseases. This process involves developing patient-derived iPSCs, transducing these cells with wild-type forms of the mutant gene, differentiating these gene-corrected cells into neuronal precursors, and transplanting them back into the patient's central nervous system (Griffin et al., 2015). This process attempts to achieve the same aim as *in vivo* gene therapy by establishing a long-term source of wild-type enzyme within the brain, but without injection of adenovirus into patients.

Recent studies have assessed the efficacy of human iPSC-derived cell transplants into mouse models of two LSDs, metachromatic leukodystrophy (MLD) and Sly disease (MPS VII). Before transplant, these LSD mice lines were crossed with immunodeficient mice to avoid immune rejection. As discussed earlier, Doerr et al. (2015) generated neuroepithelial stem cells and astroglial progenitors from MLD patient iPSCs that were transduced with a vector containing the wild-type *ARSA* and transplanted into the brains of MLD mice. This did result in a significant reduction of sulfatide in the vicinity of transplanted cells. Griffin et al. (2015) similarly transplanted neural stem cells differentiated from iPSCs from patients with Sly disease, and noted GUSB activity along with correction of disease-associated microglial pathology. These studies illustrate the success of correcting brain pathology using genetically reprogrammed iPSCs and the survival of neural stem cells and astroglial progenitors after several months. However, no experiments were performed to assess whether disease symptoms in the mice were reduced.

### iPSCs as a platform for drug screening

Small-molecule chaperones are another strategy that could be appropriate for the treatment of LSDs. Such drugs would function by binding endogenous mutant enzyme, stabilizing the protein and thereby increasing enzymatic activity. Like SRT drugs, these small molecules would be able to enter the brain, but unlike SRT drugs, they would act by directly addressing the underlying enzyme deficiency. Currently, high-throughput drug screens are commonly used. Different small-molecule libraries have been assembled containing a hundred thousand to a million compounds that can be tested simultaneously (Inglese et al., 2006; Zheng et al., 2007). Other libraries containing FDA-approved compounds are also available for such screens. Chaperones for different LSDs have been identified by employing assays that screen for compounds that impact enzymatic activity (Motabar et al., 2010). These assays were initially utilized to identify enzyme inhibitors that bind to the active site (Zheng et al., 2007). Subsequently, tissue extracts were used to identify non-inhibitory chaperones that are now being developed further (Jung et al., 2016).

iPSC-derived cell models can play a role in identification of small-molecule drugs as well as providing a new platform for testing new drugs. Although it is currently difficult to generate a large enough number of cells to use in high-throughput screening, they can still serve as a valuable validation tool for candidate drugs. In the case of GD, two different groups examined specific small-molecule inhibitors of glucocerebrosidase that act as pharmacological chaperones, and both observed improvement in the clearance of erythrocytes and reduction in the secretion of pro-inflammatory factors in iPSC-derived macrophages (Panicker et al., 2014; Tiscornia et al., 2013). Furthermore, Aflaki et al. (2014) demonstrated correction of glucocerebrosidase activity, lipid storage, chemotaxis and reactive oxygen species (ROS) production in iPSC-derived macrophages treated with a novel non-inhibitory chaperone. These results demonstrate that iPSC-derived cells provide opportunities for both the identification and documentation of responses to new therapies.

A different strategy to improve the folding of mutant lysosomal enzymes exploits proteostasis regulators. In GD, calcium channel blockers were shown to partially restore enzymatic activity in patient fibroblasts; rescue of activity was thought to involve upregulation of the intrinsic molecular chaperones of glucocerebrosidase (Wang et al., 2011). IPSC-derived models might prove useful in the identification and testing of such regulators, as well as enabling a better understanding of their mechanism of action. Therapies based on heat shock proteins are also under consideration for several LSDs (Kirkegaard et al., 2016). Theoretically, therapies combining chaperones and proteostasis regulators could enhance efficacy, and iPSCs are also an effective platform for testing and optimizing such combinatorial therapies.

## Insights into common neurodegenerative diseases

One of the most profound benefits of iPSC models is the ability to recapitulate the hallmark characteristics of cells affected by common neurodegenerative disorders. In particular, the differentiation of iPSCs into DA neurons has provided the ability to investigate the previously unattainable diseased neurons implicated in neuronopathic GD and Parkinson's disease. A complete understanding of the basis of the relationship between glucocerebrosidase and parkinsonism is still lacking (Aflaki et al., 2017), augmenting the need for new tools and models. A recent study by Woodard et al. generated iPSC-derived neuronal models from a set of monozygotic twins discordant for PD, both of whom carried an N370S mutation in *GBA1* (Woodard et al., 2014). The study revealed increased α-synuclein levels in DA neurons in the twin with Parkinson's disease. Such investigations provide a platform upon which the complex association between *GBA1* and Parkinson's disorder can be further elucidated and ultimately characterized. In another study, Aflaki et al. examined differentiated DA neurons from patients with GD1, GD1-with Parkinson's disease and GD2 (Aflaki et al., 2016a). These cells were then used to test non-inhibitory compounds that could be potential leads for drug development. Ultimately, such studies have shown that iPSC-derived neurons can circumnavigate the difficulties in studying human tissue in neurodegenerative disorders.

## Caveats and limitations of iPSC-based models for LSDs

Despite the advantages provided by iPSCs for modeling different LSDs, there are some issues that are important to take into consideration. Some of the limitations of this technology are listed in Box 2, and discussed below.

**Box 2: Limitations of iPSC-based disease models of lysosomal storage disorders**

Developing iPSC-based disease models is expensive, labor-intensive and requires time

Reprogramming is energetically demanding and can be affected by metabolic defects intrinsic to the disease being modeled

The donor cells must be carefully and completely phenotyped

Controls are needed with an appropriate genetic background

The model may not reflect later-onset disease phenotypes

iPSC-derived differentiated cells might not retain aging-associated gene signatures and cellular properties

### Metabolic impediments to reprogramming and differentiation

The process of reprogramming is energetically demanding, and cells must undergo extensive metabolic remodeling in order to successfully transition to pluripotency (Choi et al., 2015; Panopoulos et al., 2012). When generating iPSC-derived cell models from patients with LSDs, there is the possibility that the metabolic disruption, accumulation of storage material and subsequent cellular dysfunction seen in LSDs could negatively impact the reprogramming process. iPSC lines for several LSDs have exhibited extensive disease-related pathology. Although most iPSC models of LSDs have been developed without the rescue of the deficient enzyme, difficulties in reprogramming of patient cells have been reported, as highlighted in specific sections above (Huang et al., 2011; Lemonnier et al., 2011; Tiscornia et al., 2013).

### Phenocopying: do these cells provide a faithful model of disease?

Two universal metrics for assessing the effectiveness of an iPSC-derived model of LSDs are enzyme deficiency and substrate storage. Most, but not all, LSD iPSC lines have exhibited these features before differentiation; however, the presence of these defects in differentiated cells is required for them to be considered a potential model of disease. When evaluating other observed cellular phenomena, researchers generally aim to compare their findings to established pathologies in human patients or animal models, when available. Perhaps the strongest support for the effectiveness of the ability of iPSC-derived cells to phenocopy their *in vivo* counterparts was provided by the observation that macrophages differentiated from both Gaucher iPSCs and peripheral blood monocytes derived from the same patients exhibited similar cellular phenotypes (Aflaki et al., 2014).

However, in many cases, pluripotent stem cell (both ESCs and iPSC)-derived differentiated cells often best resemble cells of the early embryo (<6 weeks of development) rather than cells from adult tissues (Keller, 2005; Patterson et al., 2012). Owing to their immature state, the functionality of such cells could be different from their adult counterparts. For this reason, maturation of cells can sometimes be required, and this is achieved by supplementation of chemical compounds that promote more rapid maturation (Chambers et al., 2012). Another strategy used to generate more mature and functional pluripotent stem cell-derived cells is to try to reproduce the *in vivo* conditions by co-culturing with other cell types from the native tissue environment, such as glia cells in the case of neurons. Furthermore, three-dimensional approaches such as the generation of organoids that reproduce the organ architecture *in vitro* or by microfluidics systems (organ-on-a-chip) that are able to recreate dynamic multi-tissue structures have been considered (Cornacchia and Studer, 2017). Another limitation is that iPSC-derived differentiated cells might not retain aging-associated gene signatures and cellular properties such as senescence and proliferation, mitochondrial metabolism and related oxidative stress (Lapasset et al., 2011; Marion et al., 2009; Prigione et al., 2010; Suhr et al., 2009). This could pose a problem when studying aging-related disease pathophysiology *in vitro*, such as bone pathology in Gaucher disease. Attempting to control the cellular age of differentiated cell linages has become a major challenge, particularly when developing models of neurodegenerative diseases. For this reason, strategies aimed at modeling the effect of aging, such as treatment with ROS, or the manipulation of particular transcriptional regulators, signaling pathways and epigenetic markers are being considered (Cornacchia and Studer, 2017; Miller et al., 2013).

### Selecting donor cells

Determining which donor cells to use to model the LSDs can be impacted by the paucity of available patient samples. As a result of the rarity of these diseases, biorepositories are often the only source of fibroblasts from patients with LSDs. However, information regarding disease phenotypes can be lost when patient cells are entered into biorepositories. In the worst cases, iPSC lines can be completely misidentified. In fact, the first two GD iPSC lines were generated using the same fibroblast line from the same biorepository, but the publications disagreed over the disease phenotype of the donor (Mazzulli et al., 2011; Park et al., 2008b). More generally, a major asset of patient-derived iPSCs lies in the correlation of the iPSC phenotype with the patient phenotype, which is particularly important when considering the vast phenotypic heterogeneity that characterizes the LSDs. Studies that source fibroblasts directly from well-characterized patients are therefore particularly valuable.

### Cost

Perhaps the largest impediment to the development and use of iPSC-derived models is the cost. Firstly, reagents, media, consumables and growth factors are quite expensive. Furthermore, modeling any disease using iPSCs is labor-intensive and requires a great investment in human resources. This is compounded by the long periods of time required for the reprogramming process, iPSC validation and differentiation to relevant cell types. This is particularly true when attempting to establish adult-differentiated cells and/or to recapitulate later-onset disease phenotypes. Moreover, because these are rare diseases, it is difficult to generate a large number of LSD iPSC models with different genotypes in order to perform studies with adequately high statistical power.

### Identifying the appropriate controls

Identifying and generating appropriate controls with the same genetic background of the disease model can also be challenging. To overcome the differences in genetic background and also clonal variability, which can occur during reprogramming (Gore et al., 2011; Hussein et al., 2011), generating isogenic lines using genome-editing systems [such as transcriptional activator-like effector nucleases (TALENs), or clustered regulatory interspaced short palindromic repeat (CRISPR)/Cas-based systems] is desirable (Gaj et al., 2013). These technologies can also be used to introduce disease-specific mutations in wild-type cells in order to generate a phenotype. However, these endeavors are likely to also be challenging and labor intensive.

## Conclusion

The ability to generate iPSC models of different LSDs is markedly changing the approach to modeling these disorders. In particular, these new methods of generating diseased macrophages, neurons and cardiomyocytes closely resembling the primary disease phenotypes provide new tools to probe disease pathogenesis and to test therapeutic strategies. One issue that has remained unresolved is to what extent the phenomena observed in the disease models are physiologically relevant, as opposed to being a result of the reprogramming or differentiation process. New advances in gene editing could help to answer these questions. To confirm that changes observed in the cell models are a result of the specific mutation, TALENs and/or CRISPR-Cas strategies can now be used to correct diseased iPSCs by editing out the disease-causing mutations (Kim et al., 2017). This technology, while requiring extensive optimization, will enable researchers to ascertain what features of the cellular models are a direct functional consequence of the LSD-associated mutation.

Differentiating the iPSCs into different neuronal lineages will help to elucidate the cause of neuronopathic forms of LSDs. In addition, although a link between Gaucher disease and the synucleinopathies is clearly established, it has not been definitively ascertained whether mutations in other LSD genes are similarly related to more common neurodegenerative disorders. iPSC models of these rare, often lethal disorders could provide unique opportunities to phenotype neurons expressing the mutant lysosomal genes.

The generation of organoid disease models from iPSCs is a rapidly growing field, developed to bridge the gap between studies in cell lines and *in vivo* modeling. Such research has been supported by progress in stem cell work and in new biomaterials. This has enabled researchers to develop 3D culture systems mimicking conditions found in human tissues. Developing organoids to model the different lysosomal storage disorders is clearly of great interest, and likely to be an expanding field in the future.
